# A cytosol-tethered YHB variant of phytochrome B retains photomorphogenic signaling activity

**DOI:** 10.1007/s11103-024-01469-2

**Published:** 2024-06-14

**Authors:** Wei Hu, J. Clark Lagarias

**Affiliations:** Department of Molecular and Cellular Biology, University of California, 1 Shields Avenue, Davis, CA 95616 USA

**Keywords:** Light-independent phyB signaling, Cytoplasmic phytochrome signaling, Photomorphogenesis, Subcellular localization, Plant photoreceptors

## Abstract

The red and far-red light photoreceptor phytochrome B (phyB) transmits light signals following cytosol-to-nuclear translocation to regulate transcriptional networks therein. This necessitates changes in protein–protein interactions of phyB in the cytosol, about which little is presently known. Via introduction of a nucleus-excluding G767R mutation into the dominant, constitutively active *phyB*^*Y276H*^ (*YHB*) allele, we explore the functional consequences of expressing a cytosol-localized YHB^G767R^ variant in transgenic Arabidopsis seedlings. We show that YHB^G767R^ elicits selective constitutive photomorphogenic phenotypes in dark-grown *phyABCDE* null mutants, wild type and other phy-deficient genotypes. These responses include light-independent apical hook opening, cotyledon unfolding, seed germination and agravitropic hypocotyl growth with minimal suppression of hypocotyl elongation. Such phenotypes correlate with reduced PIF3 levels, which implicates cytosolic targeting of PIF3 turnover or PIF3 translational inhibition by YHB^G767R^. However, as expected for a cytoplasm-tethered phyB, YHB^G767R^ elicits reduced light-mediated signaling activity compared with similarly expressed wild-type phyB in *phyABCDE* mutant backgrounds. YHB^G767R^ also interferes with wild-type phyB light signaling, presumably by formation of cytosol-retained and/or otherwise inactivated heterodimers. Our results suggest that cytosolic interactions with PIFs play an important role in phyB signaling even under physiological conditions.

**Supplementary Information:**

The online version contains supplementary material available at 10.1007/s11103-024-01469-2.

## Key message

Cytoplasmic phytochrome B activity is revealed by expression of a cytosol-tethered, constitutively active YHB^G767R^ mutant allele.

**Supplementary Information:**

The online version contains supplementary material available at 10.1007/s11103-024-01469-2.

## Introduction

The plant phytochrome (phy) family are reversibly photochromic dimeric biliproteins that sense the red and far-red light spectrum of the ambient environment to regulate optimal growth and development (Rockwell et al. 2006; Franklin and Quail 2010). Light absorption by the covalently attached bilin chromophore triggers conformational changes that underlie reversible photoisomerization of phys between their red-absorbing, physiologically inactive Pr and far-red-absorbing, active Pfr states. Within the phy family in *Arabidopsis thaliana*, phyA and phyB play major roles in seedling photomorphogenesis—phyA mediates very-low-fluence responses and the high-irradiance response to far-red light, whereas phyB mediates low-fluence (red/far-red reversible) responses. Our understanding of phy signaling has been greatly aided by the identification of gain-of-function (GOF) alleles. The most well studied of such alleles encodes the missense Y276H variant of Arabidopsis phyB (YHB) (Fischer and Lagarias 2004; Su and Lagarias 2007). YHB is poorly photoactive and strongly fluorescent, and *YHB*-expressing transgenic plants exhibit dominant constitutive photomorphogenesis (*cop*) phenotypes (Su and Lagarias 2007; Hu et al. 2009). Since YHB function is light independent, *YHB*-expressing plants have widely been used to interrogate phyB signaling without activation of other light-dependent processes (Galvao et al. 2012; Jung et al. 2016; Huang et al. 2019; Alves et al. 2020; Hu et al. 2020; Chen et al. 2022).

Canonical early phyB signaling mechanisms have been revealed through three decades of studies. Newly synthesized phyB holoprotein resides in the cytosol as the inactive Pr form. The photoconversion from Pr to Pfr unmasks an intrinsic, yet undetermined, nuclear localization signal within its C-terminal half that permits its interaction with nuclear transport facilitators and subsequent cytosol-to-nucleus translocation (Chen et al. 2005; Pfeiffer et al. 2012). Nuclear Pfr-phyB aggregates as discrete photobodies most notable at high fluences of red light (Yamaguchi et al. 1999; Chen et al. 2022). Such photobodies become the orchestrating hub through which phyB interacts with an array of transcription factors such as PHYTOCHROME-INTERACTING FACTORs (PIFs) and other signaling components to profoundly rewire the transcriptional regulation of photomorphogenesis (Ni et al. 1998; Leivar and Quail 2011; Cheng et al. 2021; Kim et al. 2023). In the nucleus, Pfr-phyB induces rapid phosphorylation and consequent ubiquitin-26S proteasome pathway-mediated degradation of PIFs to abolish their growth-promoting and skotomorphogenesis-sustaining functions (Al-Sady et al. 2006; Leivar et al. 2008; Shin et al. 2009; Ni et al. 2017; Pham et al. 2018). Nuclear import thus appears to be a prerequisite for phyB to execute these regulatory roles (Huq et al. 2003; Fankhauser and Chen 2008; Klose et al. 2015). Consistent with its constitutive signaling activity, YHB always forms a few large nuclear photobodies even in the absence of light (Su and Lagarias 2007; Chen et al. 2010).

During dark-to-light transitions, nascent cytosolic Pfr-phy species must disengage from cytosolic retention complexes and productively engage with nuclear translocation factors—processes that are poorly understood. Both phenomena likely impact the observed cytosolic signaling responses reviewed by Hughes (2013). These include phy-mediated (1) transient increase in cytosolic Ca^2+^ levels (Shacklock et al. 1992), (2) interaction with cytosolic protein PENTA1 to suppress the translation of protochlorophyllide reductase (*PORA*) mRNA (Paik et al. 2012), and (3) interaction with phototropins at the plasma membrane to modulate directional response to light and gravity (Rosler et al. 2010; Jaedicke et al. 2012). More recently, the acute red-light dependent spike of cytosolic Ca^2+^ levels was shown to activate Ca^2+^-dependent kinases that in turn phosphorylate Pfr-phyB and promote phyB nuclear import during early de-etiolation transition (Zhao et al. 2023). Whereas these phenomena are most evident during seedling de-etiolation, their contributions to de novo phyB-mediated seedling photomorphogenesis are difficult to distinguish from processes affected by photosynthesis.

To address the importance of cytoplasmic signaling functions of phyB, we introduced a loss-of-function (LOF) G767R mutation into the constitutively active *YHB* allele. Among the strongest known *PHYB* LOF missense alleles, the phyB^G767R^ variant is defective in nuclear import and exhibits imperceptible light-signaling activity (Wagner and Quail 1995; Matsushita et al. 2003; Pfeiffer et al. 2012). Addition of a nuclear localization signal (NLS) to a phyB^G767R^-GFP chimera not only confers phyB^G767R^ nuclear localization in darkness, but also restores its photoregulatory activity (Matsushita et al. 2003). Based on this evidence, we reason that the YHB^G767R^ protein will adopt a ‘constitutively active’ state (like YHB) without being translocated to the nucleus. The present studies address the physiological consequence of YHB^G767R^ expression in the *phyABCDE* null mutant of Arabidopsis to test the effects of sustained cytosolic activation of phyB in darkness while also avoiding interference from photosynthesis and/or potential protein–protein interactions with other phys. We also examine the influence of YHB^G767R^ expression on light-grown seedling development in wild-type and various *phy* mutant backgrounds in which photosynthesis is restored. These studies provide novel insights into phyB-dependent processes in the cytoplasm which influence signaling pathways occurring in the nucleus.

## Materials and methods

### Constructs and transgenic plants

The G767R mutation was introduced into *YHB* and *PHYB* genomic sequences in the pJM78 plasmid (Su and Lagarias 2007) by site-directed mutagenesis using primers 5ʹ-GTCGGCGTTTGTTTTGTT*C*GACAAGACGTTACTAGTC-3ʹ and 5ʹ-GACTAGTAACGTCTTGTC*G*AACAAAACAAACGCCGAC-3ʹ. Upon sequencing validation, the *YHB*^*G767R*^ and *PHYB*^*G767R*^ inserts were excised with *Sac*II and *Pst*I, and subcloned into the pJM63-PHYB^g^ binary vector (Su and Lagarias 2007) (Fig. S1A). pJM63-YHB^g^-G767R was then transformed into the *phyABCDE* null mutant, the *phyB-5* mutant and the L*er* WT using the floral dip method (Clough and Bent 1998; Hu et al. 2013; Jones et al. 2015). In addition, pJM63-PHYB^g^-G767R was transformed into the *phyB-5* mutant, and pJM63-PHYB^g^ was transformed into the *phyABCDE* mutant. Standard genetic procedures were employed to secure multiple single-insertion, homozygous transgenic lines. *YHB*^*g*^/*phyABCDE* and *YHB*^*g*^/*phyB-5* were derived from *YHB*^*g*^/*phyA-201phyB-5* line #5 by outcrossing (Su and Lagarias 2007; Hu et al. 2009; Jones et al. 2015). *35S::YHB/phyB-5* #10 was described previously (Su and Lagarias 2007). The pBI-*pPHYB:PHYB*^*C357S*^*/phyB-1* (No-0 ecotype) was a generous gift from Prof. Robert Sharrock (Montana State University) (Clack et al. 2009). All wild type and other transgenic lines were in the L*er* ecotype background.

### Growth conditions and phenotypic analyses

**Fig. 1:**
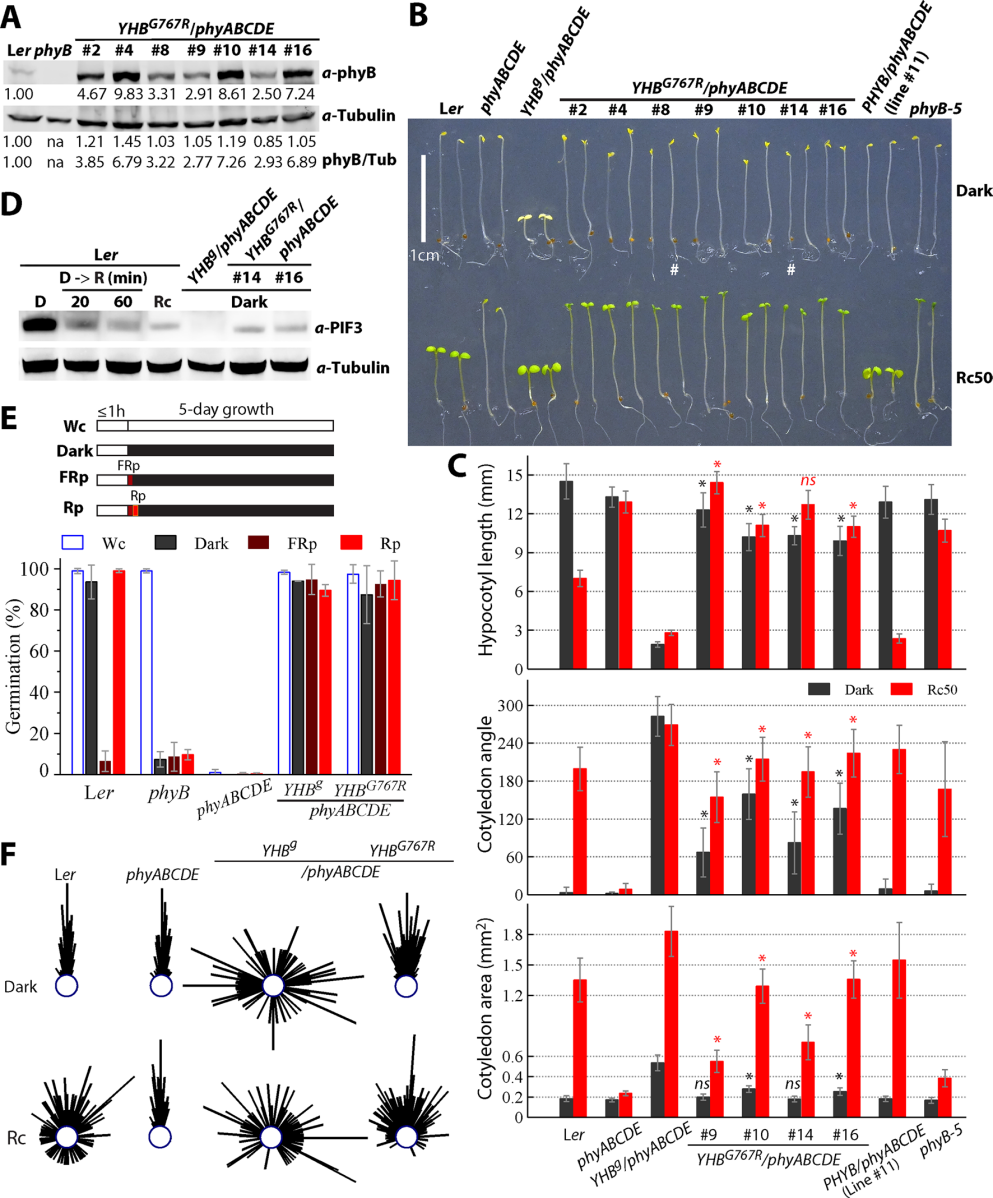
The G767R mutation strongly, yet incompletely suppresses the constitutive activity of YHB in the *phyABCDE* mutant background. **A** Immunoblot analyses of *YHB*^*G767R*^*/phyABCDE* lines. **B** Phenotypic comparison of 4-day-old seedlings grown in true darkness or under continuous red light (50 µmol m^−2^ s^−1^); # denotes *YHB*^*G767R*^ seedlings with etiolated morphology. **C** Quantification of hypocotyl lengths, cotyledon separation angles and cotyledon areas of two overexpressed *YHB*^*G767R*^*/phyABCDE* lines (#10, #16) and two less expressed lines (#9, #14) in comparison to other control genotypes; *denotes statistical significance compared to *phyABCDE* grown under the same condition (*p* < 0.0001; Student’s t-test), *ns*, not statistically significant; *n* =  ~ 30. **D** Immunoblot analyses of dark-grown seedlings reveal significant, but not complete loss of PIF3 protein by YHB^G767R^ in the overexpressing line #16 and the less expressing line #14. **E** YHB^G767R^ promotes light-independent seed germination; six transgenic lines were used for *YHB*^*G767R*^ and three replicates for other genotypes, each replicate used ~ 100 seeds. **F** Circular histograms depict seedling growth directions in darkness or under continuous red light (Rc50); YHB^G767R^ data were from two independent lines, *n* =  ~ 100

Seeds were surface sterilized with 75% ethanol for 12 min, then suspended with 0.1% phytagar and sown on 1 × MS medium (pH 5.7, 0.8% phytagar). Following 4-day stratification, plates were exposed to ≥ 3 h white light (~ 80 µmol m^−2^ s^−1^) to induce synchronized germination. Seedlings were grown in continuous red light (Rc, 50 µmol m^−2^ s^−1^), or in ‘true darkness’ where plates were additionally exposed to 5 min of FR pulse (20 µmol m^−2^ s^−1^) before wrapping with aluminum foil (Leivar et al. 2008). Seedlings were scanned or photographed and measured digitally in the NIH ImageJ software (https://imagej.nih.gov/ij/). For seed germination assays, approximately 100 seeds of each genotype for each replicate experiment were surface sterilized and sown on 0.75% phytagar plates (pH 5.7) within an hour and then allowed to germinate within a five-day period in darkness, under continuous cool fluorescent white light (~ 50 µmol m^−2^ s^−1^), exposed to 5 min far-red light pulses (FRp, 12 µmol m^−2^ s^−1^) before darkness, or exposed to additional 5 min red light pulses (Rp, 20 µmol m^−2^ s^−1^) following FRp before placing in darkness (Fig. 1D). Seeds were deemed germinated if the radicle emerged. For seedling growth direction quantification, plates were placed vertically; the growth angles from 100 seedlings of each genotype measured in ImageJ were plotted as circular histograms.

### Immunoblot assay and confocal fluorescence microscopy

Protein extraction and immunoblot assays were performed as previously described (Su and Lagarias 2007; Jones et al. 2015; Hu and Lagarias 2017). Blot intensities were quantified using LI-COR Image StudioLite software (for phyB and tubulin) or ImageJ (for PIF3). Confocal fluorescence microscopy was performed as described previously (Hu and Lagarias 2017).

### Subcellular fractionation and immunoblot assay

Overexpression *PHYB/phyABCDE* line #3 and *YHB*^*G767R*^*/phyABCDE* line #2 were used for fractionation. Approximately 10–12 leaves of short day-grown (8 h L/16 h D), 50-d-old plants were collected at mid-day to isolate mesophyll protoplasts using a slightly modified *Tape-Arabidopsis-Sandwich* protocol (Wu et al. 2009). Leaves peeled off of the abaxial epidermis were incubated in 8 ml of digestion enzyme solution with gentle shaking for an hour under ambient room light. Released protoplasts were collected by centrifugation (100 g × 3 min) and rinsed three times with W5 solution; after the final spin, the protoplasts were gently resuspended in 1 ml pre-cooled NIBA buffer (CelLytic™ PN Isolation/Extraction Kit, Sigma-Aldrich; Cat. # CELLYTPN1). Good harvest of intact protoplasts was confirmed by observation under light microscope. Protoplasts were lysed by adding 0.3% (v/v, final) Triton X-100 followed by incubation on ice for 10 min with intermittent gentle shaking. Lysates were spun at 12,000 g × 10 min to separate cytoplasmic (supernatant) and nuclear (pellet) fractions. Supernatant proteins were purified using the hot SDS extraction buffer followed by methanol-chloroform purification (Su and Lagarias 2007). Nuclear pellets were resuspended in 1 ml NIBA buffer and re-centrifuged. The re-centrifuged nuclear pellets were then lysed with 200 µl hot SDS extraction buffer followed by methanol-chloroform protein purification (Su and Lagarias 2007). Total protein was extracted from aliquots of protoplasts as described previously (Su and Lagarias 2007). Protein was quantified by BCA assay, and 50 µg of each sample were loaded for SDS-PAGE. Mouse anti-RNA Polymerase II (RPII) monoclonal antibody (Abcam, cat. #ab5408) was used in 1/1000 dilution for blotting. Immunoblotting procedure and antibodies against phyB and Actin were as described previously (Jones et al. 2015; Hu and Lagarias 2017).

## Results

### *YHB*^*G767R*^* retains selective light-independent signaling activity in the phyABCDE null mutant background*

To explore the extent of suppression by the G767R mutation on the constitutively active *YHB* allele, we introduced both Y276H and G767R missense mutations into the genomic *PHYB* expression construct *pJM63-PHYB*^*g*^ (Fig. S1A) (Su and Lagarias 2007) and transformed the resultant double mutant construct into the *phyABCDE* null mutant (Hu et al. 2013). Immunoblot assay of seven *YHB*^G767R^/*phyABCDE* lines revealed a range of expression from 2.8- to 6.9-fold that of the endogenous phyB level (Fig. 1A). We also transformed the null mutant with the *pJM63-PHYB*^*g*^ construct as a wild-type (WT) phyB-only control, recovering six highly overexpressing *pJM63-PHYB*^*g*^/*phyABCDE* lines and one line with ~ threefold endogenous expression level (Fig. S1B, C). Despite the use of the native *PHYB* promoter, our observed overexpression results agree with previous studies showing that the strong promoter/enhancer driving expression of the selectable marker frequently overrides the specificity of the transgene promoter residing within the same construct (Yoo et al. 2005; Hu and Lagarias 2017).

In contrast to *YHB*-expressing lines that exhibit background-independent strong *cop* phenotypes (Su and Lagarias 2007; Hu and Lagarias 2017), all *YHB*^*G767R*^/*phyABCDE* lines grown for 4 days in true darkness possessed elongated hypocotyls, confirming that the G767R mutation suppressed the GOF signaling activity of YHB on hypocotyl growth (Fig. 1B, C). However, dark-grown *YHB*^*G767R*^/*phyABCDE* seedlings lacked apical hooks and possessed open cotyledons, in contrast to dark-grown L*er* WT, *phyABCDE* null, *phyB-5* and *PHYB*-complemented *phyABCDE* seedlings, all of which retained apical hooks and closed cotyledons (Fig. 1B, C). These results indicate that YHB^G767R^ selectively retains GOF signaling activity.

Compared to the etiolated phenotype of *phyABCDE* null mutants under 50 µmol m^−2^ s^−1^ continuous red light (Rc50), *YHB*^*G767R*^/*phyABCDE* seedlings had fully opened and green cotyledons despite retaining elongated hypocotyls (Fig. 1B, C). Such seedlings had larger cotyledons than Rc-grown *phyB-5* single mutants, and the two overexpressed lines #10 and #16 were even comparable to Rc-grown WT—a result indicating significant rescue of R-dependent photomorphogenesis to the null mutant (Fig. 1C). A transgene dosage effect was observed among Rc-grown seedlings, with higher YHB^G767R^ expressing lines having larger cotyledons, e.g. #2, #4, #10 and #16 vs #8, #9 and #14 (Fig. 1B, C). These results show that although the G767R mutation nearly eliminates YHB inhibition of hypocotyl growth under red light, significant red light-dependent photomorphogenesis is retained in the *YHB*^*G767R*^ transgenic seedlings.

Previous studies have established that the protein level of the PIF3 transcription factor in dark-grown seedlings is a sensitive molecular indicator for YHB function (Hu and Lagarias 2017). We therefore examined the PIF3 levels present in the *YHB*^*G767R*^/*phyABCDE* seedlings by immunoblot analysis. Representative high and relatively low *YHB*^*G767R*^-expressing lines, i.e. #16 and #14 respectively, both exhibited greatly reduced PIF3 levels that were more similar to those of Rc-grown WT than dark-grown WT (Fig. 1D). These results suggest that YHB^G767R^, like YHB and photoactivated WT phyB, elicits the loss of PIF3 protein.

Other YHB-conferred traits include light-independent seed germination and randomized growth direction both in darkness and under red light. We therefore examined the influence of YHB^G767R^ on these two developmental responses in the *phyABCDE* background. On phytagar medium lacking mineral nutrients, WT L*er* showed R/FR-reversible seed germination and *phyB-5* only germinated efficiently under Wc, while *phyABCDE* failed to germinate under all light conditions (Fig. 1E) unless GA was added (Hu et al. 2013). By contrast, seeds of both YHB and YHB^G767R^ lines germinated well in darkness and under all light conditions (Fig. 1E). Hypocotyls of WT Arabidopsis seedlings display negative gravitropism (upward growth) in darkness, whereas their growth is randomized, or agravitropic, under red and far-red light—a response known to be mediated by multiple phytochromes (Robson and Smith 1996; Kim et al. 2011). As expected, *phyABCDE* mutants exhibited constitutive negative gravitropism and *YHB* seedlings grew with randomized orientation both in the dark and under Rc (Fig. 1F). By comparison, the growth direction of dark-grown *YHB*^*G767R*^ seedlings were more random than those of L*er* and *phyABCDE*, but still were largely negatively gravitropic. Under Rc, *YHB*^*G767R*^ seedlings exhibited greatly randomized growth, although its orientation range was less than those of L*er* and YHB (Fig. 1F). These results show that YHB^G767R^ also retains signaling activity to promote light-independent seed germination and to inhibit negative gravitropism.

### *YHB*^*G767R*^* is retained in the cytoplasm*

**Fig. 2:**
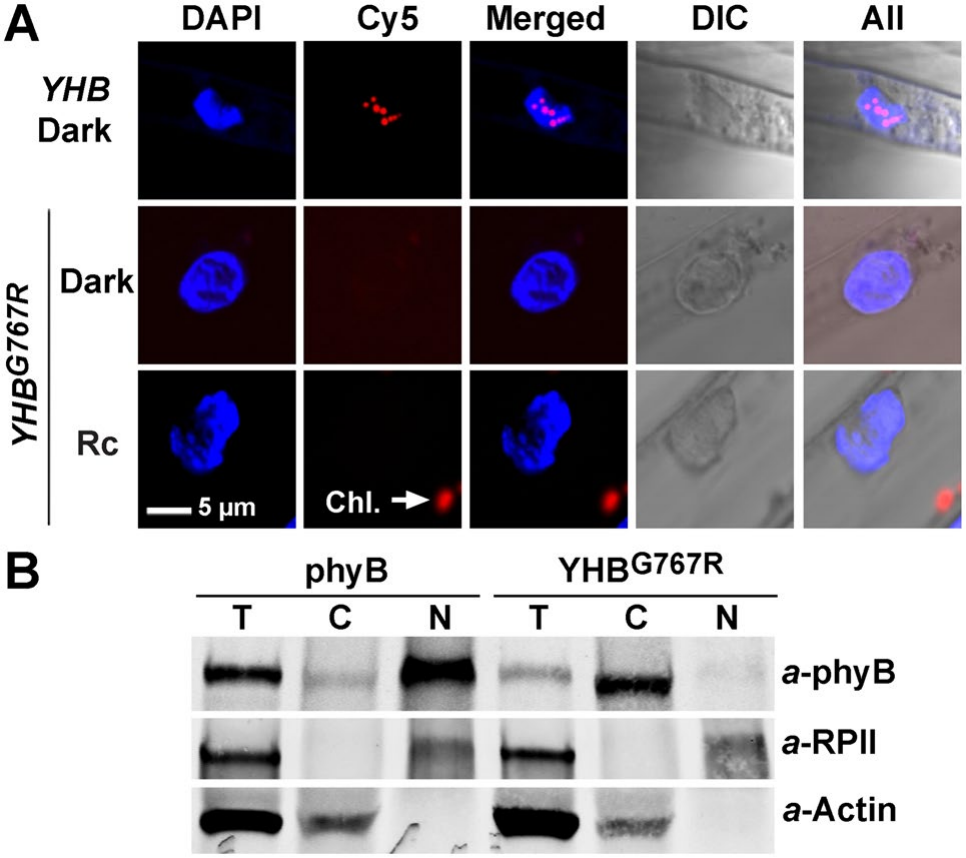
YHB^G767R^ is localized in cytoplasm. **A** Confocal fluorescence microscopy reveals the absence of nuclear photobodies in *YHB*^*G767R*^/*phyABCDE* grown in the dark or under continuous red light, whereas *YHB*/*phyABCDE* displays steady nuclear photobodies; Chl., chlorophyll autofluorescence. **B** Fractionation immunoblotting reveals cytosolic distribution of YHB^G767R^ from light-grown transgenic plants. T, total soluble protein; C, cytoplasmic fraction; N, nuclear fraction. RPII, RNA Polymerase II

Previous studies have established that the G767R mutation inhibits light-dependent nuclear localization of phyB while not altering its chromophorylation, photochemistry, dark reversion or dimerization (Wagner and Quail 1995; Matsushita et al. 2003; Shin et al. 2011). This inability to migrate into the nucleus accounts for the loss of function of phyB^G767R^, since addition of an NLS restores the nuclear localization and photoregulatory activity of a GFP-fusion of this LOF allele (Matsushita et al. 2003). We reasoned that introduction of the G767R mutation into YHB might similarly affect its subcellular localization while not altering its chromophorylation or light-independent activation. Since Arabidopsis YHB is a red fluorescent protein and constitutively forms a few large nuclear photobodies independent of light conditions (Su and Lagarias 2007; Hu and Lagarias 2017), we took advantage of confocal microscopy to probe the localization of YHB^G767R^. Unlike the constitutive nuclear localization pattern of YHB (Fig. 2A), no red fluorescent signal was detected from nuclei of *YHB*^*G767R*^-expressing seedlings grown in the dark or under Rc. These results indicate that YHB^G767R^ is not imported into the nucleus. To corroborate this interpretation, we performed fractionation immunoblot assays for light-grown plants. Using photoactivated phyB as the positive control for the nuclear fractions, the assays showed that YHB^G767R^ was only detectable in the cytosolic fraction and absent from the nuclear fraction (Fig. 2B). Therefore, the G767R mutation inhibits nuclear migration of YHB, analogous to its effect on WT phyB. Taken together, these results strongly suggest that the cytoplasmic signaling activity of YHB^G767R^ accounts for its influence on seedling photomorphogenesis shown in Fig. 1.

### *Both YHB*^*G767R*^* and PHYB*^*G767R*^* can complement phyB mutants under red light in a dosage-dependent manner*

**Fig. 3:**
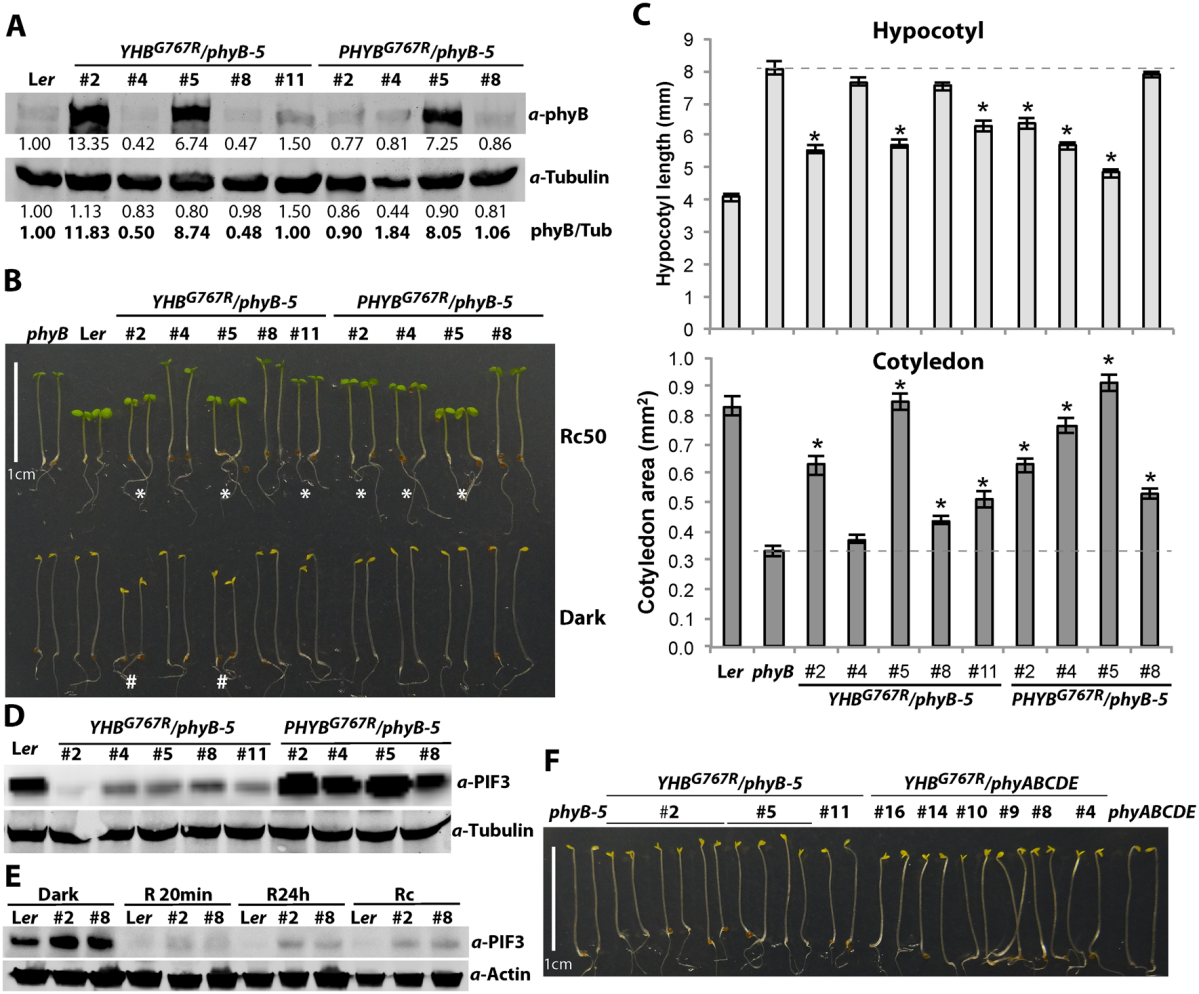
YHB^G767R^ and PHYB^G767R^ complement the *phyB-5* mutant in a dosage-dependent manner. **A** Immunoblot analysis of nine genetically single-insertion homozygous transgenic lines. **B** Four-day-old seedlings grown in continuous red light (50 µmol m^−2^ s^−1^) or in darkness. * denotes transgenic lines with discernable shorter hypocotyls than *phyB-5* mutants; #denote lines with discernable *cop* phenotype in the dark. **C** Quantification (mean ± S.D.) of hypocotyl lengths (top) and cotyledon sizes (bottom) of Rc50-grown seedlings; * denotes statistical significance in comparison to *phyB-5* (*p* < 0.01; Student’s t-test), *n* = 20. **D** Immunoblot analysis of PIF3 protein levels in dark-grown seedlings. **E** Immunoblot analysis of red light-induced PIF3 loss in *PHYB*^*G767R*^/*phyB-5* lines #2 and #8 with comparable expression levels to endogenous phyB. **F** Phenotypic comparison of true dark-grown *YHB*^*G767R*^ transgenics in the *phyB-5* and *phyABCDE* mutant backgrounds

To examine the signaling activities of YHB^G767R^ and phyB^G767R^ in the presence of other phys, we next introduced their corresponding genomic constructs into the *phyB-5* single mutant. Immunoblot analysis revealed that some of these lines had expression levels similar to or even lower than that of endogenous phyB (Fig. 3A). In darkness, all of the *PHYB*^*G767R*^ lines remained fully etiolated, while only the most strongly overexpressing *YHB*^*G767R*^ lines (#2 and #5) exhibited partial GOF hypocotyl growth suppression and cotyledon opening (Fig. 3B, bottom). By comparison, *YHB*^*G767R*^ lines with endogenous expression levels (#11) or lower (#4 and #8) displayed marginal to undetectable cotyledon opening, with no significant influence on hypocotyl growth (Fig. 3B, bottom). Whereas all dark-grown *YHB*^*G767R*^ lines had greatly reduced PIF3 levels, PIF3 abundance remained high in the *PHYB*^*G767R*^ lines (Fig. 3D). These results corroborate the observed GOF activity of YHB^G767R^ in the *phyABCDE* null background and also show that phyB^G767R^ is signaling inactive in darkness as expected.

Under Rc, both *YHB*^*G767R*^ and *PHYB*^*G767R*^ alleles were able to significantly complement the *phyB-5* mutant; however, only overexpressing lines were able to phenocopy WT as evaluated by hypocotyl lengths, cotyledon sizes, or both (Fig. 3B upper and 3C). The effect of R light on PIF3 levels in two *PHYB*^*G767R*^ lines with endogenous expression levels (#2 and #8; see Fig. 3A) was compared with WT L*er*. After 20 min of R-light exposure on dark-grown seedlings, both WT and *PHYB*^*G767R*^ lines experienced significant PIF3 loss, consistent with its light-dependent turnover (Fig. 3E). The R-dependent loss of PIF3 was nearly complete in L*er*, whereas both *PHYB*^*G767R*^ lines still retained some residual PIF3 levels. This reduction of PIF3 levels also was sustained following prolonged R-light exposure where the role of phyA is suppressed by its turnover (Fig. 3E, R24h and Rc). Indeed, these data corroborate those of a previous study which established that overexpression of *PHYB*^*G767R*^ was able to complement the *phyA-211;phyB-9* double mutant (Col accession) under Rc, ruling out a role for phyA in the sustained loss of PIF3 (Park et al. 2018). Overall, our results support the hypothesis that cytosol-tethered phyB^G767R^, like YHB^G767R^ in darkness (Fig. 3C), can trigger PIF3 turnover and/or inhibit PIF3 translation under Rc. Despite the great loss of PIF3 in all Rc-grown *PHYB*^*G767R*^ and *YHB*^*G767R*^ lines, overexpression is required to rescue seedling photomorphogenesis of the *phyB-5* mutant.

Finally, we also performed a side-by-side comparison of true dark-grown *YHB*^*G767R*^/*phyB-5* and *YHB*^*G767R*^/*phyABCDE* lines. Since no discernable difference was observed in YHB^G767R^-dependent weak *cop* phenotypes, the presence of other phys does not appreciably modulate YHB^G767R^ function in darkness (Fig. 3F).

### *Dosage-dependent effect of YHB*^*G767R*^* in the presence of functional phyB alleles*

**Fig. 4:**
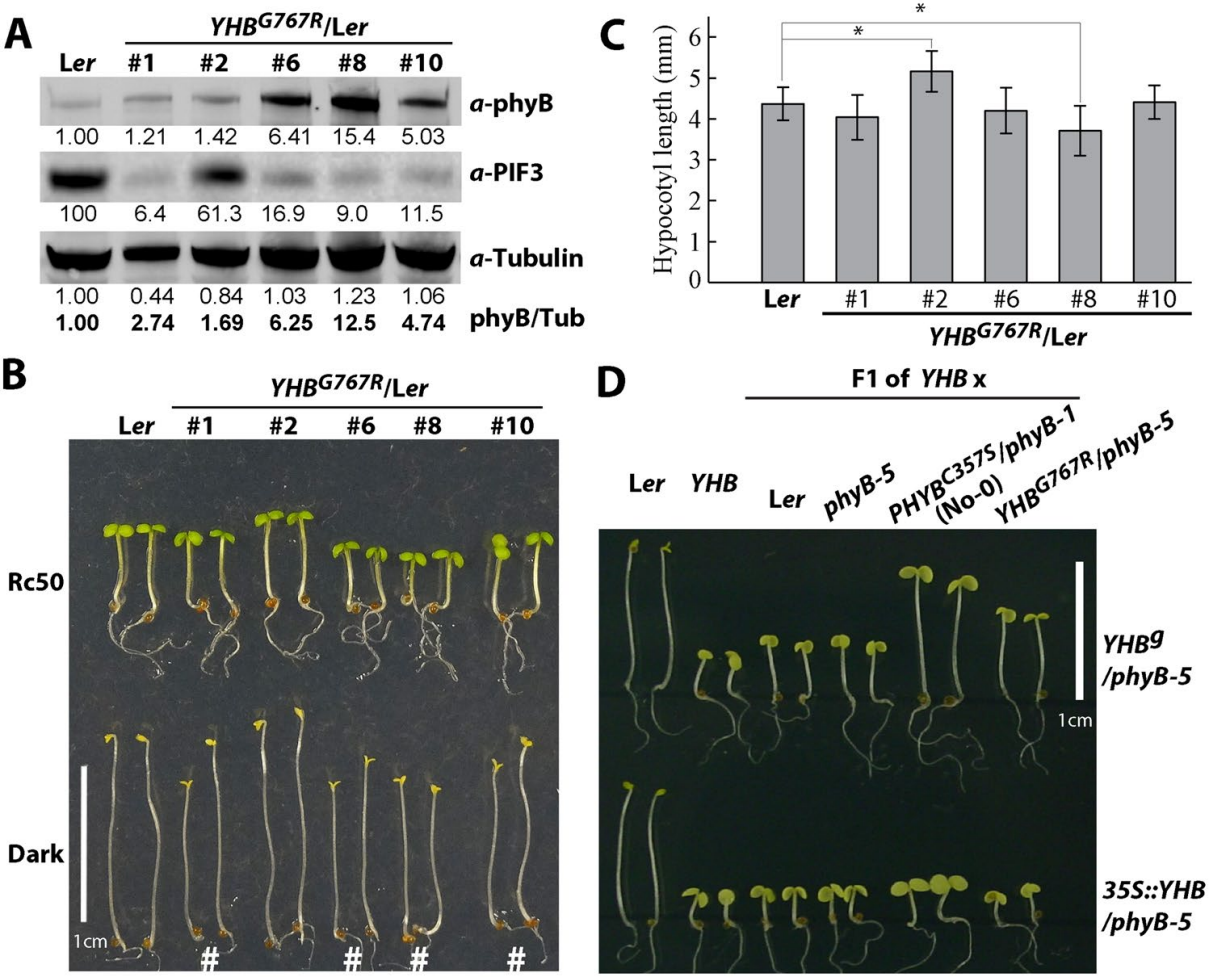
Dosage-dependent effects of YHB^G767R^ in the presence of functional phyB alleles. **A** Immunoblot analysis of dark-grown seedlings. **B** Four-day-old seedlings grown in continuous red light (50 µmol m^−2^ s^−1^) or in the dark; # denotes lines with discernable *cop* phenotypes (partially opened cotyledons). **C** Hypocotyl lengths (mean ± S.D.) of Rc50-grown, 4-d-old seedlings; * denotes statistical significance in comparison to L*er* (*p* < 0.001; Student’s t-test), *n* = 20. **D** Dark-grown F1 seedlings of genetic crosses between a weak-expressing *YHB*^*g*^/*phyB-5* or an overexpressing *35S::YHB*/*phyB-5* line with L*er* WT, *phyB-5* and two cytoplasm-retained *PHYB* mutation lines

*YHB*^*G767R*^ was also introduced into L*er* WT to test its activity in the presence of WT phyB, and lines with varied transgene expression levels were obtained (Fig. 4A). In lines #1, #6, #8 and #10, in which YHB^G767R^ accumulated to levels higher than endogenous phyB, PIF3 levels in dark-grown seedlings were drastically reduced, whereas a significant amount of PIF3 protein was detected in the lowest expressing line #2 (Fig. 4A). The four moderately and highly expressing YHB^G767R^ lines also displayed weak *cop* phenotypes in the dark, whilst line #2 did not (Fig. 4B). When grown under Rc, only line #8 with the highest YHB^G767R^ expression (more than ten-fold of endogenous phyB) exhibited statistically significant inhibition of hypocotyl growth, whereas line #2 exhibited the opposite trend with more elongated hypocotyls (Fig. 4B, C). Because of the dimerization nature of phyB, we propose that formation of cytosol-retained YHB^G767R^:phyB heterodimers might be responsible for these dosage-dependent observations.

To further test this hypothesis, we crossed both low- and high-expressing *YHB* lines, i.e. *YHB*^*g*^ and *35S::YHB,* respectively, with L*er* WT, the *phyB-5* mutant, and *YHB*^*G767R*^- and *PHYB*^*C357S*^-expressing lines (Fig. 4D). *YHB*^*g*^ outcrosses with either L*er* WT or *phyB-5* yielded F1 seedlings with slightly longer hypocotyls in darkness, consistent with the expected decrease in *YHB*^*g*^ transgene dosage. By contrast, *YHB*^*g*^ outcrosses with *YHB*^*G767R*^ or with chromophore-less *PHYB*^*C357S*^-expressing lines significantly promoted F1 seedling elongation, suggesting that both cytosol-retained phyB variants dampen nuclear import of some YHB molecules by heterodimerization (Fig. 4D). As predicted, neither variant proved effective in suppressing the GOF activity of overexpressed YHB, presumably due to the presence of saturating levels of YHB:YHB homodimers in these lines (Fig. 4D).

### *Genetic background independence and dosage dependence of YHB*^*G767R*^* function*

**Fig. 5:**
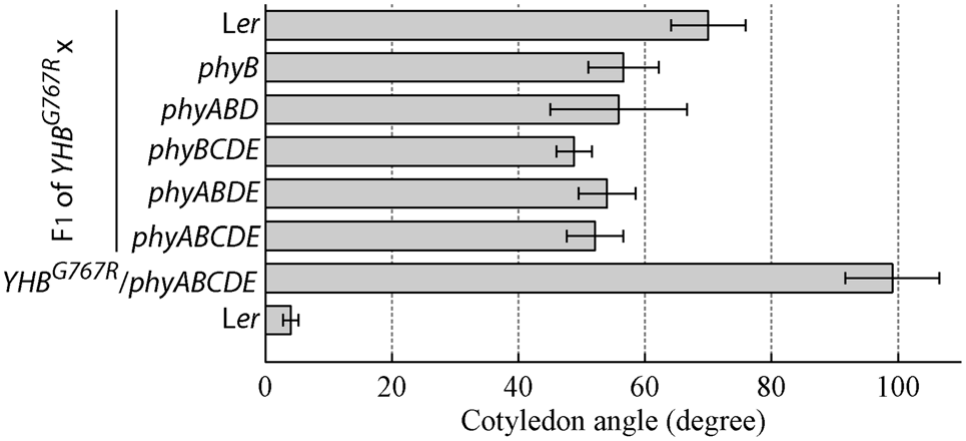
Effects of YHB^G767R^ dosage and genetic backgrounds on seedling cotyledon opening in true darkness, mean ± s.e.m., *n* = 17 ~ 22. *YHB*^G767R^/*phyABCDE* line #16 was used for outcrosses with Ler WT and various *phy* mutants

Finally, to further confirm that the photomorphogenesis-promoting role of YHB^G767R^ is dosage-dependent and largely independent of the presence of other phys, we outcrossed the overexpressing *YHB*^*G767R*^/*phyABCDE* line #16 with L*er* WT and with other *phy* mutants. Consistent with the decrease by half of the YHB^G767R^ levels in various F1 seedlings harboring a *phyB* mutation, cotyledon opening angles of these dark-grown F1 seedlings were reduced to half the degrees seen in the parental *YHB*^*G767R*^/*phyABCDE* line (Fig. 5). Interestingly, F1 seedlings resulting from the outcross with L*er* WT exhibited slightly greater cotyledon angles than those F1 seedlings retaining a *phyB* mutant allele, which also may reflect the effect of increased number of dimers containing YHB^G767R^ by heterodimerization with phyB. Overall, this outcross experiment supports that the YHB^G767R^ signaling activity is dosage-dependent and independent of the presence of other non-phyB phys.

## Discussion

The G767R mutation has long been known to profoundly suppress phyB signaling function (Wagner and Quail 1995; Matsushita et al. 2003). Since this mutation is located outside the N-terminal photosensory module (PSM) of the full-length photoreceptor, it does not alter the photochemical property of phyB in vivo or in vitro (Wagner and Quail 1995; Shin et al. 2011). Instead, this mutation inhibits phyB nuclear import (Matsushita et al. 2003; Pfeiffer et al. 2012). Our confocal microscopy and subcellular fractionation work also establishes that YHB^G767R^ is retained in the cytosol (Fig. 2). For this reason, the *cop* phenotypes of dark-grown YHB^G767R^-expressing Arabidopsis seedlings likely reflect the signaling action of a cytosol-localized phyB species. Beyond the limits of the detection sensitivity of our measurement, however, we cannot rule out the possibility that a trace portion of YHB^G767R^ might reside in the nucleus exerting the observed signaling function. Given the significant loss of PIF3, strong seed germination de-repression and cotyledon opening, our data support a light-independent cytosolic action of YHB^G767R^ in these photomorphogenetic responses.

YHB^G767R^/*phyABCDE* enables studying the function of cytosolic photoactive phyB without interference from endogenous phy signaling or from light-triggered physiological activities when grown in darkness. Although earlier work using dark-grown *YHB*^*G767R*^/*phyABCDE* demonstrated that this cytosolic phyB allele could not maintain circadian robustness (Jones et al. 2015), the present studies show that dark-grown *YHB*^*G767R*^/*phyABCDE* seedlings display partial de-etiolation, notably hook opening and cotyledon expansion, and reduced gravitropic growth (Fig. 1). Moreover, *YHB*^*G767R*^ transgenic lines exhibit light-independent seed germination and PIF3 protein loss (Fig. 1). These observations imply that nuclear translocation is not essential for these signaling aspects of photoactivated phyB.

Overexpressed tagged PIF3 was observed to diffusely distribute in the nucleus of dark-grown transgenic plants (Bauer et al. 2004; Al-Sady et al. 2006) and also in nuclei of transfected onion epidermal cells (Ni et al. 1998). Upon red light irradiation, YFP-PIF3 swiftly forms nuclear photobodies, followed by rapid phosphorylation and turnover—a process mainly mediated by phyA. Such PIF3 behavior is consistent with the light-induced rapid nuclear import and subsequent turnover of phyA (Al-Sady et al. 2006). PIF3 is known to interact with both N- and C-terminal regions of phyB, showing much stronger interaction with the N-terminal PSM of photoactivated phyB than with the C-terminal regulatory domain (Ni et al. 1999). In contrast to FHY1/FHL-mediated nuclear translocation of photoactivated phyA (Hiltbrunner et al. 2005, 2006), nuclear-targeting of photoactivated phyB is less well understood. We know that the C-terminal fragment of phyB is constitutively localized to nuclear bodies (Yamaguchi et al. 1999), that phyB nuclear targeting requires the C-terminal regulatory domain of the photoreceptor (Chen et al. 2005), and that early nuclear photobody formation of phyB requires the presence of PIF3 (Bauer et al. 2004). PIF3 was also shown to be needed to mediate full-length phyB import into the nuclei of the model green alga *Acetabularia*, and the G767R mutation prevented PIF3-induced nuclear import of the C-terminal phyB fragment (Pfeiffer et al. 2012). Together with the evidence that other PIF proteins influence the nuclear accumulation of phyB-YFP chimera (Pfeiffer et al. 2012) and that PIF3 was initially identified using a yeast two hybrid screen with the C-terminal phyB fragment (Ni et al. 1998), these studies support that PIF3 and other PIFs mediate phyB nuclear translocation via interaction with the C-terminus of phyB in a light-dependent manner. Consistent with this hypothesis, removal of the C-terminal region of phyB prevents its nuclear translocation *in planta*, despite the known strong light-dependent interaction between PIFs and the N-terminal PSM of phyB (Ni et al. 1999; Matsushita et al. 2003).

Based on this knowledge, we propose that PIFs interact with cytosol-constrained YHB^G767R^ via its N-terminal PSM that has been constitutively activated by the Y276H mutation. We further speculate that a significant portion of PIF3 resides in the cytoplasm of dark-grown seedlings, poised to interact with newly photoactivated phyB to implement early nuclear import of phyB-PIF3 complex. That fluorescent protein tagged PIF3 was not reported to be seen in the cytoplasm likely is due to spatial dilution of such chimera protein in the vast cytosolic volume in comparison to the nucleus. We conclude that YHB^G767R^-induced PIF3 loss occurs in the cytosol, as cytosol-retained YHB^G767R^ cannot physically interact with nuclear PIF3. The smaller amount of PIF3 detected in dark-grown *YHB*^*G767R*^ lines may be those comparted in the nucleus and inaccessible to YHB^G767R^.

The observed reduction of PIF3 levels in dark-grown *YHB*^*G767R*^-expressing seedlings suggests that YHB^G767R^ either targets PIF3 for degradation and/or inhibits translation of the *PIF3* mRNA in the cytosol. The first scenario implies the residual PIF3 would be nuclear localized, whereas the latter scenario implies it would be cytosolic. The latter possibility already has been reported for the *PORA* transcript (Paik et al. 2012), so the potential role of cytosolic phyB inhibiting PIF3 translation remains a viable hypothesis. Photoactivated phyB, likely of the nuclear portion, has been shown to enhance retention of an intron in the *PIF3* 5’UTR to inhibit PIF3 protein translation (Dong et al. 2020). Based on these considerations, measurement of residual PIF3 localization in these plants will not resolve this issue. Although definitive resolution of the two mechanistic hypotheses is beyond the scope of this study, we note that PIF3 transcript levels are not significantly altered in dark-grown *YHB*-expressing plants compared with WT L*er* (Hu et al. 2009). This result argues that the inhibition of *PIF3* transcription unlikely accounts for the reduced PIF3 protein levels in dark-grown *YHB*^*G767R*^ plants.

Given that cytosolic photoexcited phyB rapidly induces transient spike of cytosolic Ca^2+^ levels during seedling de-etiolation (Zhao et al. 2023), it is not surprising some phy signaling cascades occur in the cytoplasm (Hughes 2013). Indeed, in addition to PIF3 turnover, our studies show that light-independent seed germination is also elicited by cytosolic YHB^G767R^. Since PIF1 is a key negative regulator of seed germination, which is targeted for turnover by photoactivated phyB (Oh et al. 2004, 2006; Shen et al. 2005), PIF1 is likely to be degraded by YHB^G767R^ in the cytoplasm as well. This interpretation is supported by the work of Park et al. (2018) showing that PIF1 levels are greatly reduced in red light-grown *PHYB*^*G767R*^/*phyA-211;phyB-5* seedlings. Their results and our own data argue that the loss of PIF1 is caused by photoactivated, cytosol-tethered phyB. Based on these results, models of phyB action may need to consider the role of cytosolic interactions between phyB and PIFs in the regulation of gene expression.

In summary, YHB^G767R^ is a valuable germplasm to study signaling actions of cytosolic phyB in both darkness and the presence of light. Our studies demonstrate that the GOF activities of YHB^G767R^ in various *phyB* mutant backgrounds exhibit a protein dosage dependence. We also show that G767R alleles of phyB can suppress the regulatory activities of other phytochromes likely through heterodimerization. Whereas it is clear that maximal phyB signaling in developing seedlings requires nuclear translocation, our studies argue that cytosolic signaling by phyB must not be discounted even under physiological light conditions. Moreover, the relatively small effect of YHB^G767R^ on hypocotyl growth suppression suggests that cytosolic interactions with PIFs and possibly other factors that influence seed germination, hook-opening and cotyledon development take precedence to processes such as hypocotyl, stem and root growth regulated by other PIFs, such as PIF4 and PIF5. This makes sense since rapid elongation growth is needed for the radicle to breach the seed coat during germination. The recent reports that PIF3 plays a critical role in root penetration into soil via interacting with the cytosolic transmembrane receptor FERONIA (Xu et al. 2024), and that FERONIA can phosphorylate phyB (Liu et al. 2023), suggest that the interplay between phyB, PIF3 and FER in the cytosol are important for early seedling establishment in Arabidopsis.

### Supplementary Information

Below is the link to the electronic supplementary material.

Supplementary file1 (DOCX 568 KB)

## Data Availability

The data supporting the findings of this study are available within the paper and its Supplementary Information, and also from the authors upon reasonable request.
