# Supplementary material for: A cytosol-tethered YHB variant of phytochrome B retains photomorphogenic signaling activity
Source: Plant Mol Biol. 2024 Jun 14;114(4):72. doi: 10.1007/s11103-024-01469-2 (PMC11178650; doi:10.1007/s11103-024-01469-2)
Supplement: Supplementary file 1 — Supplementary file1 (DOCX 568 KB) [file 11103_2024_1469_MOESM1_ESM.docx]

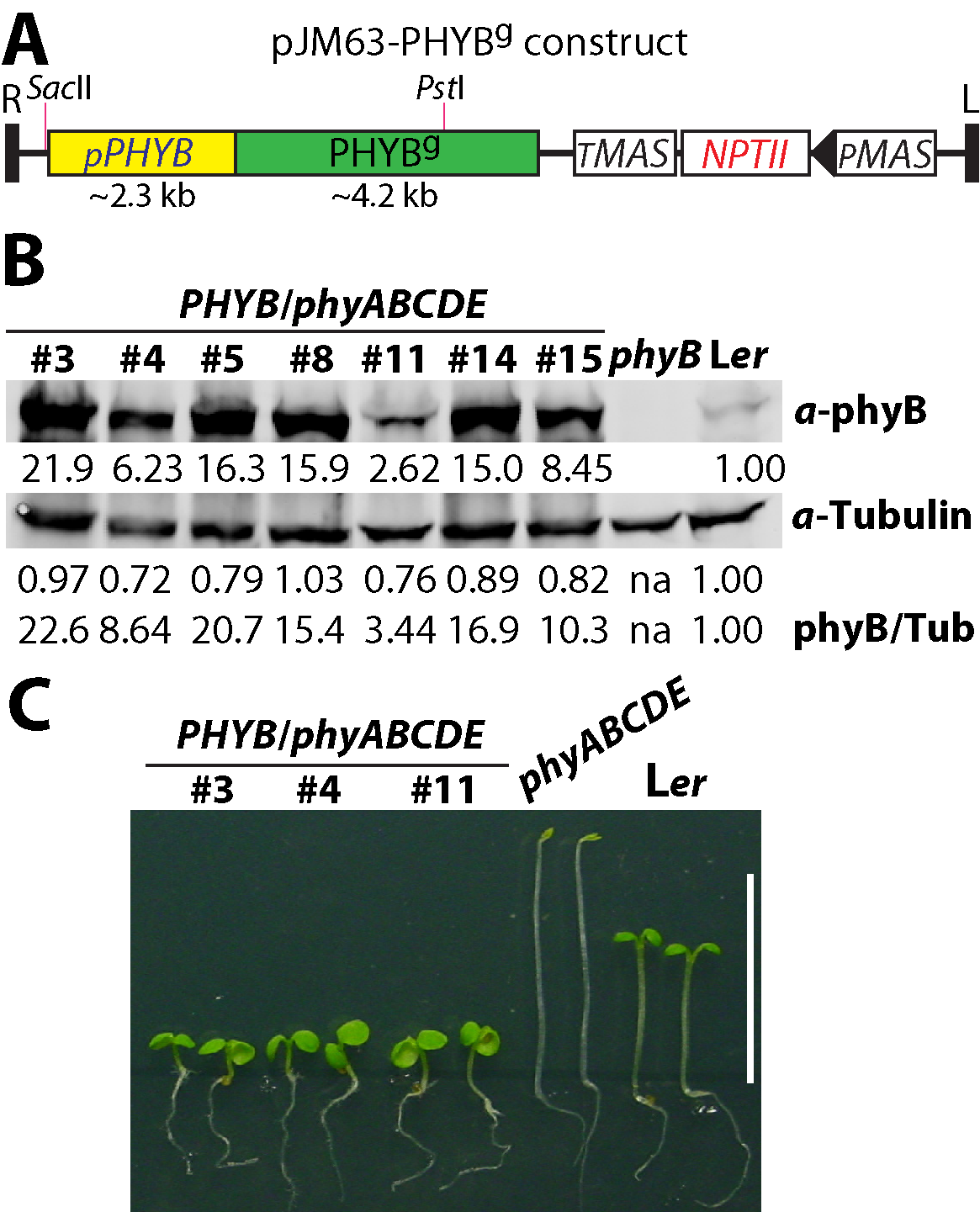


**Supplementary Fig. 1** The pJM63-PHYB^g^ construct frequently has overexpression effect in transgenic plants because of the overriding effect from the flanking strong *MAS* promoter driving the *NPTII* selectable marker. (A) Diagram of the pJM63-PHYB^g^ vector (T-DNA region); *Sac*II and *Pst*I restriction sites for subcloning are indicated; PHYB^g^, genomic PHYB fragment including introns and 3’UTR; *MAS*, mannopine synthase. (B) Immunoblot analysis of seven *pJM63-PHYB^g^/phyABCDE* transgenic lines; line #11 has the lowest phyB level but is still higher than the endogenous phyB level. (C) Four-day-old seedlings grown under continuous red light (50 µmol m^-2^ s^-1^); three *PHYB/phyABCDE* lines representing varied transgene expression levels exhibit similar light hypersensitive growth phenotypes.
